# Calcio-herbal medicine Divya-Swasari-Vati demonstrates acceptable non-clinical safety profile in the repeated-dose 28-day subacute oral toxicity study in Sprague-Dawley rats, under GLP compliance

**DOI:** 10.3389/fphar.2025.1547532

**Published:** 2025-06-17

**Authors:** Acharya Balkrishna, Sandeep Sinha, Anurag Varshney

**Affiliations:** ^1^ Drug Discovery and Development Division, Patanjali Research Foundation, Haridwar, India; ^2^ Department of Allied and Applied Sciences, University of Patanjali, Haridwar, India; ^3^ Patanjali UK Trust, Glasgow, United Kingdom; ^4^ Special Centre for Systems Medicine, Jawaharlal Nehru University, New Delhi, India

**Keywords:** 28-day repeated-dose toxicity, Divya-Swasari-Vati, OECD 407, GLP, Ayurveda

## Abstract

**Introduction:**

Ayurvedic medicines with scientifically proven anti-inflammatory, bronchodilator, anti-tussive and anti-microbial activities have an immense potential to be repurposed for the management of obstructive airway symptoms, frequently encountered in patients afflicted with coronavirus disease 2019 (COVID-19). Divya-Swasari-Vati is an Ayurvedic prescription medicine, which contains nine botanical drugs and seven mineral ashes. The individual botanical drugs and minerals present in Divya-Swasari-Vati have been traditionally used for the treatment of upper and lower respiratory tract symptoms, associated with ailments ranging from common cold and cough to chronic asthma and respiratory infections. Divya-Swasari-Vati is enriched with metabolites known to possess pharmacological activity and has demonstrated *in-vivo* anti-inflammatory effects in a human A549 cell line-xenotransplanted zebrafish model, subsequent to challenge with the spike protein of severe acute respiratory syndrome-coronavirus-2. With an objective to support the preclinical and clinical profile, the non-clinical safety assessment of Divya-Swasari-Vati is highly warranted. Accordingly, in the current study, we report the non-clinical safety of Divya-Swasari-Vati in a repeated-dose, 28-day subacute oral toxicity study, followed by a 14-day recovery period, in Sprague-Dawley rats, under GLP compliance.

**Methods:**

This toxicological study was conducted according to Organization for Economic Cooperation and Development (OECD) test guideline 407 and in conformance with the OECD principles of Good Laboratory Practice (GLP). Divya-Swasari-Vati was tested at the doses of 100, 300 and 1,000 mg/kg/day, in five males and five female rats of each experimental group.

**Results:**

In the present study, no mortality or morbidity was observed in any of the test groups. Furthermore, Divya-Swasari-Vati treatment was not associated with any toxicologically relevant outcomes with respect to clinical signs as well as clinical-, gross- and histo-pathological findings, as compared to the vehicle-administered group. Consequently, the No-Observed-Adverse-Effect-Level (NOAEL) of Divya-Swasari-Vati was determined to be 1,000 mg/kg/day, in both male and female rats.

**Discussion:**

The acceptable safety profile of Divya-Swasari-Vati demonstrated in the present study, provisions for its future non-clinical safety assessments for longer duration in rodents as well as in higher animals. Additionally, this study also serves as the first step towards the detailed assessment of Divya-Swasari-Vati in clinical settings.

## 1 Introduction

Divya-Swasari-Vati (DSV) is a calcium-enriched Ayurvedic prescription medicine, which comprises of the fine powders of nine botanical drugs along with seven mineral ashes referred to as *Bhasmas* (Kumar et al., 2006), in the Ayurvedic system of medicine. The botanical drugs present in DSV include the roots of *Glycyrrhiza glabra* L. and *Anacyclus pyrethrum* (L.) Lag; the galls of *Pistacia chinensis* subsp. *Integerrima* (J. L. Stewart) Rech. f.; the fruits of *Cressa cretica* L., *Piper longum* L. and *Piper nigrum* L.; the rhizomes of *Zingiber officinale* Roscoe; the bark of *Cinnamomum verum* J. Presl and the flower buds of *Syzygium aromaticum* (L.) Merr. and L. M. Perry. Apart from these botanical drugs, seven mineral *Bhasmas* are also present in DSV, namely, Mukta-Shukti *Bhasma*, Godanti *Bhasma*, Kapardak *Bhasma*, Abhrak *Bhasma*, Sphatika *Bhasma*, Praval *Pishti* and Tankan *Bhasma*. The botanical drugs and mineral ashes present in DSV, their description and their relative share in the medicine have been listed in Table 1, in alphabetical order. DSV has been distinctly formulated by incorporating the traditional wisdom of Ayurveda, as a potential remedy for the treatment of various lung diseases. These etiologies range from mild upper airway symptoms to progressive life-threatening pneumonia, which are witnessed in a majority of symptomatic patients, infected with the severe acute respiratory syndrome-coronavirus 2 (SARS-CoV2) (Ackermann et al., 2020). The progressive decline in lung function has been observed, especially in institutionalized coronavirus disease 2019 (COVID-19) patients. It is directly attributed to the release of pro-inflammatory signaling molecules from the host cells and other cellular components of the host defense system. These pro-inflammatory molecules orchestrate the late stage development of pulmonary edema, thickening of the alveolar interstitium and augmented vascular permeability (Wiersinga et al., 2020). Consequently, modulating the underlying inflammation, which is closely associated with the disease progression, is expected to improve the therapeutic outcomes. In this light, the positive clinical data of the broad spectrum anti-inflammatory agent dexamethasone in reducing the 28-day patient mortality, substantiates the repositioning of existing anti-inflammatory therapies for the treatment of airway obstruction associated with COVID-19 (Tomazini et al., 2020). Moreover, targeted immunomodulatory therapies like tocilizumab (Salama et al., 2021) and sarilumab (Della-Torre et al., 2020), which are anti–interleukin-6 receptor monoclonal antibodies; anakinra, which is a recombinant human interleukin-1 receptor antagonist (Aouba et al., 2020) and ruxolitinib, which is Janus kinase 1 and 2 dual-inhibitor (Cao et al., 2020) have also been evaluated for the management of COVID-19. However, use of these targeted therapies may compromise the patient’s immune system and consequently lead to secondary infections (Khiali et al., 2020; Balkrishna et al., 2022). Conversely, medicines derived from botanical drugs, can potentially exert favorable effects by acting as prophylactic as well as therapeutic agents by inhibiting viral attachment, genome replication and anti-inflammatory effects (Boozari and Hosseinzadeh, 2021). To exemplify, multistep molecular docking simulations have revealed that plant metabolites could bind to Nsp-15, a SARS-CoV2 endoribonuclease, which hampers recognition of the viral pathogen by host cells (Parihar et al., 2023). Furthermore, plant metabolites can also bind to two of the most crucial proteins of SARS-CoV2 involved in viral replication, namely, RNA-dependent RNA polymerase and the main protease of the virus (Parihar et al., 2022).

**TABLE 1:** Composition of Divya-Swasari-Vati (DSV) powder.

Botanical drugs (as fine powders)					
Sr No.	Botanical name [Family]	Hindi vernacular name	English name	Part used	Relative share in DSV (%)
1	*Anacyclus pyrethrum* (L.) Lag. [Asteraceae]	Akarkara	Spanish chamomile	Root	5.92
2	*Cinnamomum verum* J. Presl [Lauraceae]	Dalchini	Cinnamon	Bark	5.92
3	*Cressa cretica* L. [Convolvulaceae]	Rudanti	Cretan alkaliweed	Fruit	11.66
4	*Glycyrrhiza glabra* L. [Fabaceae]	Mulethi	Licorice	Root	11.85
5	*Piper longum* L. [Piperaceae]	Chhoti Pipal	Long pepper	Fruit	7.77
6	*Piper nigrum* L. [Piperaceae]	Marich	Black pepper	Fruit	7.77
7	*Pistacia chinensis* subsp. *Integerrima* (J. L. Stewart) Rech. f. [Anacardiaceae]	Kakdasinghi	Zebrawood	Gall	11.66
8	*Syzygium aromaticum* (L.) Merr. and L. M. Perry [Myrtaceae]	Lavang	Clove	Flower bud	5.92
9	*Zingiber officinale* Roscoe [Zingiberaceae]	Sounth	Ginger	Rhizome	7.77
Mineral components (*Bhasmas* as fine powders)					

	Sanskrit Name	Description			
10	Abhrak *Bhasma*	Ash from calcined mica			2.33
11	Godanti *Bhasma*	Ash from rich gypsum			2.33
12	Kapardak *Bhasma*	Ash from calcined cowry shell of *Cypraea moneta*			2.33
13	Mukta-Shukti *Bhasma*	Ash from calcined shell of pearl oyster *(Pinctada fucata)*			2.33
14	Praval *Pishti*	Coral calcium powder processed with rose water			2.33
15	Sphatika *Bhasma*	Ash from calcined form of alum			2.33
16	Tankan *Bhasma*	Ash from calcined borax			2.33

Excipients used: Gum acacia, hydrated magnesium silicate and colloidal silicon dioxide. The voucher specimens of the botanical drugs were deposited and authenticated by Raw Materials Herbarium and Museum, Delhi, a division of Council of Scientific and Industrial Research–National Institute of Science Communication and Policy Research (CSIR–NIScPR), Government of India. The Family of the botanical drugs has been mentioned within the square brackets.

The rationale for positioning DSV in the therapeutic armamentarium for COVID-19 symptoms is derived from the scientifically demonstrated anti-inflammatory activities of the botanical drugs and mineral *Bhasmas* present in it (Balkrishna et al., 2020). Further, these botanical drugs and minerals have been used in Ayurveda for a long time for treating dry and productive cough, common cold, bronchitis, asthma, and respiratory infections. Of these, *G. glabra* L., whose principal metabolite is glycyrrhizin, is known to demonstrate anti-inflammatory, antioxidant, antiviral and antimicrobial activities (Pastorino et al., 2018). Another rare Himalayan botanical drug, *P. chinensis* subsp. *Integerrima* (J. L. Stewart) Rech. f. whose metabolite is a triterpene, pistagremic acid, has exhibited *in vivo* pharmacological effects in animal models of asthma, in addition to its validated anti-inflammatory and antimicrobial activities (Ahmad et al., 2020). Further, the halophytic botanical drug *C. cretica* L., which is a source of several metabolites including quercetin and rutin, has been reported to possess anti-inflammatory, antibacterial and antitussive activities (Priyashree et al., 2010). An additional botanical drug present in DSV is *Z. officinale* Roscoe, which contains pharmacologically active metabolites like gingerols and shogaols. It has well established immunomodulatory, anti-inflammatory, antimicrobial and antioxidant properties (Ali et al., 2008). *Piper longum* L. and *P. nigrum* L., the additional botanical drugs present in DSV are known to contain numerous essential oils and alkaloids like piperine, which have been shown to possess anti-inflammatory, antioxidative and antimicrobial properties (Salehi et al., 2019). Further, *C. verum* J. Presl, which is also present in DSV has proven antimicrobial, antioxidant and anti-inflammatory activities that can be ascribed to the presence of metabolites, namely, trans-cinnamaldehyde, eugenol, and linalool as well as cinnamic acid (Ranasinghe et al., 2013). Another botanical drug present in DSV is *A. pyrethrum* (L.) Lag., which contains metabolites like N-isobutyldienediynamide and polysaccharides and has been proven to demonstrate anti-inflammatory and antioxidant activities (Manouze et al., 2017). *Syzygium aromaticum* (L.) Merr. and L.M.Perry is also present in DSV and it has been demonstrated to exhibit antiviral, antimicrobial, anti-inflammatory and antioxidant activities, which can be attributed to the presence of metabolites like eugenol (Chaieb et al., 2007). Apart from these botanical drugs, the mineral components of DSV are also known to possess anti-inflammatory and antitussive properties (Balkrishna et al., 2020).

With an objective to confirm the reproducibility of the pharmacological studies on DSV, as mandated by the “Consensus statement on the Phytochemical Characterization of Medicinal Plant extracts” (ConPhyMP) (Heinrich et al., 2022), a systematic analytical process has been optimized, directed towards a reliable detection and quantification of the metabolites present in DSV, across the various manufactured batches (Balkrishna et al., 2021c; 2021d). DSV is enriched with several metabolites, such as eugenol, glycyrrhizin, piperine, gallic acid, 6-gingerol, ellagic acid, methyl gallate, ellagic acid and cinnamic acid (Balkrishna et al., 2021d). As already stated, all of these metabolites have been shown to possess anti-inflammatory activities. Since DSV is comprised of botanical drugs, whose metabolites are known to exert anti-inflammatory activities, the pharmacological effects of DSV were also evaluated in a zebrafish model (Balkrishna et al., 2020). In this humanized model, human lung A549 cells were xenotransplanted in the swim bladder of zebrafish and to mimic the SARS-CoV2 infection, the recombinant spike protein of the virus was subsequently injected. In this experiment, the administration of the recombinant spike protein triggered pathological changes like alterations in swim bladder cytology, intrusion of inflammatory cells, hemorrhage of the skin and an augmentation of behavioral fever. Administration of DSV to these diseased zebrafish demonstrated reversal of the pathophysiological alterations and in addition, it also suppressed the levels of interleukin-6 and tumor necrosis factor-α. The subsequent *in-vitro* mechanistic studies revealed that DSV could obviate the entry of SARS-CoV2 pseudovirus into human alveolar epithelial cells by interfering with the spike protein-ACE2 interaction. Further, it could also blunt the elevated cytokine response induced by different mutants of the spike proteins in alveolar epithelial cells (Balkrishna et al., 2022). Finally, Divya-Swasari-Vati when combined with other Ayurvedic interventions could reduce the time to recovery in asymptomatic and mildly symptomatic COVID-19 patients (Balkrishna et al., 2021a) and had beneficial outcomes on patient’s psychological health and quality of life (Balkrishna et al., 2021b).

Given, the established pharmacological effects of DSV, it is rather important to ascertain its non-clinical safety, in order to support its clinicotherapeutic potential in the human subjects. Accordingly, in the present study, we determined the tolerability of DSV after repeated daily oral administration to male and female Sprague Dawley rats for a period of 28-consecutive days. A satellite group of animals, which were administered with the high dose of DSV were observed for a treatment free-period of an additional 14-day to evaluate the reversibility, persistence or delayed occurrence of toxic effects. The study was conducted to provide information on major toxic effects, target organs and No-Observed-Adverse-Effect-Level (NOAEL) of DSV in rats, for establishing safety criteria. This study was performed in accordance with OECD test guideline 407 (Organization for Economic Co-Operation and Development, 1995) and in compliance with the OECD Principles of GLP.

## 2 Materials and methods

### 2.1 Test item, chemicals and reagents

Divya-Swasari-Vati (Batch number #B SWV174), a light brown free-flowing powder (produced for granulation and subsequent compression into tablets) was sourced from Divya Pharmacy, Haridwar India, where it was manufactured according to the WHO-CoPP standards of good manufacturing practices. The voucher specimens of the individual botanical drug components were deposited and subsequently authenticated by the Raw Materials Herbarium and Museum, Delhi (RHMD), India. RHMD has been established by the Council of Scientific and Industrial Research–National Institute of Science Communication and Policy Research (CSIR–NIScPR), Government of India. The individual botanical drugs present in DSV were authenticated on the basis of macroscopic studies of the submitted samples, followed by a detailed scrutiny of literature and finally matching the sample with the authentic samples deposited at RHMD. The selected metabolites in DSV were detected in the range of 1.245% w/w by utilizing ultra-high performance liquid chromatography (UHPLC). For oral administration, DSV was formulated as a suspension by using 0.5% methylcellulose as the suspending agent. DSV was found to be stable in the suspension for 24 h at room temperature, as confirmed by UHPLC (Table 2). The analytical methods employed for the quantification of metabolites as well as the stability analysis have been previously reported (Balkrishna et al., 2021d). The other reagents and chemicals used in this experiment were of the highest commercial grade.

**TABLE 2:** Stability analysis of DSV suspension. Concentration of the metabolites in a 100 mg/mL suspension of DSV in 0.5% methylcellulose, immediately after preparation and after maintaining it for 24 h at room temperature.

S. No.	Metabolite name	Chemical structure	Concentration (mg/mL) immediately after preparation	Concentration (mg/mL) after 24 h at room temperature
1	Eugenol	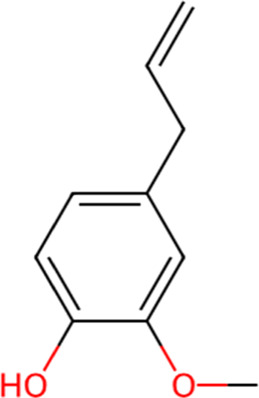	0.585	0.535
2	Glycyrrhizin	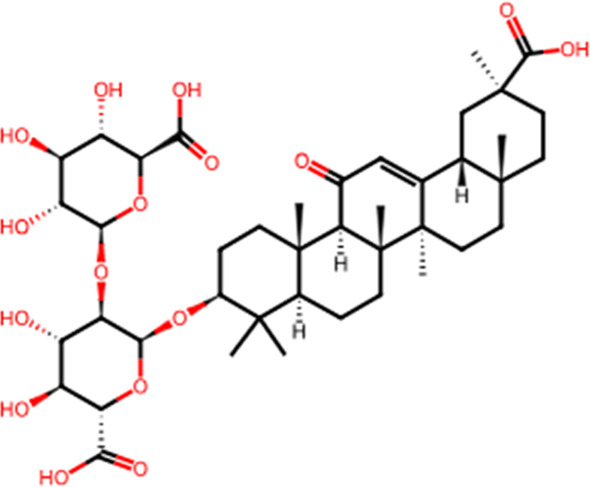	0.402	0.383
3	Piperine	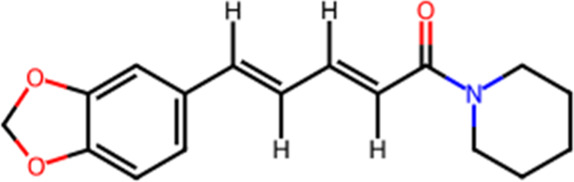	0.289	0.273
4	Gallic Acid	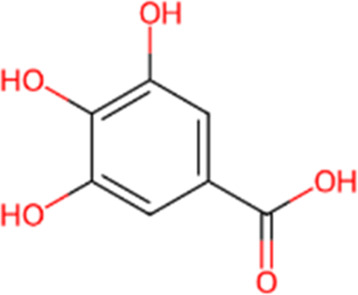	0.238	0.201
5	6-Gingerol	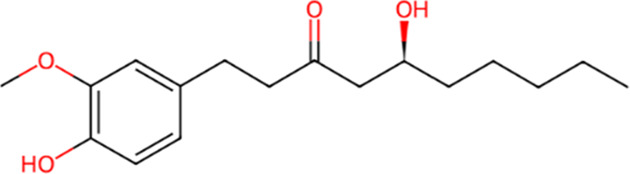	0.047	0.045
6	Ellagic Acid	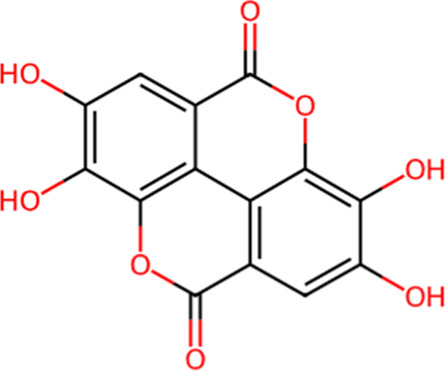	0.007	0.006
7	Cinnamic Acid	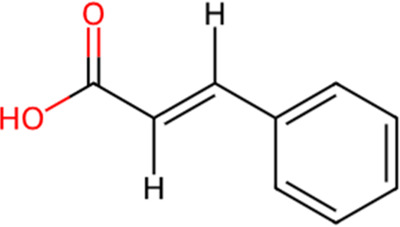	0.004	0.003

The chemical structures have been sourced from www.chemspider.com

### 2.2 Test facility for assessing the 28-day subacute toxicity of DSV

This OECD 407-driven toxicity study was performed at Preclinical Research and Development Organization (PRADO), Private Limited, Pune, India, in compliance with the OECD Principles on GLP. The test facility is certified (GLP/C-127/2018; GLP/C-168/2021) by the National GLP Compliance Monitoring Authority (NGCMA), Department of Science and Technology, Government of India, for compliance to OECD-GLP; and by Committee for the of Control and Supervision of Experiments on Animals (CCSEA; Registration number: 1723/PO/RcBiBt/S/13/CPCSEA), Department of Animal Husbandry and Dairying, Ministry of Fisheries, Animal Husbandry and Dairying, Government of India, for conducting experiments on small laboratory animals. DSV was assigned a code and provided to the test facility to ensure that the study could be conducted in a single blinded manner.

### 2.3 Experimental animals and husbandry practices

The study was conducted on 7–8 weeks old specific-pathogen-free male and female Sprague Dawley (SD) rats, which were purchased from Hylasco Biotechnology (India) Private Limited, Telangana, India; a Charles River Laboratories licensed domestic animal supplier. Rats were chosen for this study as they are one of the recommended rodent species by regulatory guidelines for the conduct of safety and toxicity studies, owing to the availability of vast historical data (Organization for Economic Co-Operation and Development, 1995). All procedures of the study as well as animal care were in accordance with the guidelines established by the CCSEA (CPCSEA, 2018) and were approved by the Institutional Animal Ethics Committee (IAEC) of PRADO (vide IAEC protocol number: IAEC-20–045). Five animals per cage and per gender were housed in sterilized individually ventilated cages (41.0 cm × 41.0 cm × 78.0 cm), that utilize the exhaust ventilation technology (Optirat^®^ Plus, Animal Care Systems, Inc., United States). Cage rotation was done at weekly intervals to ensure identical illumination and similar environment to all animals employed in the study, throughout the experiment period. Sterilized corn cob was used as the bedding material. The temperature maintained in the animal room throughout the study was 20.8°C–22.9°C and the relative humidity varied from 40% to 64%. Further, the air changes in the animal experimentation room were maintained at 10 to 15 per hour, throughout the in-life phase of the study. Additionally, the photoperiod maintained was a 12-h light-dark cycle, with the light hours being controlled by an automated Digital Electric Timer (Frontier Digital, India). Animals were supplied *ad libitum* with ultraviolet light sterilized standard pelleted laboratory animal diet (PMI-Nutrition International, LLC, New York, United States), and ultraviolet light irradiated water, purified by a reverse osmosis plant, in autoclaved polypropylene bottles. Subsequent to the receipt at the test facility, the animals were quarantined in a dedicated room for a period of 5 days.

### 2.4 Subacute toxicity study

The subacute toxicity evaluation of DSV was conducted according to OECD test guideline number 407 (Organization for Economic Co-Operation and Development, 1995).

#### 2.4.1 Animal acclimatization

After completion of the quarantine period, a total of 64 Sprague Dawley rats (32 males and 32 females) were issued by Animal Research Facility at PRADO for this study and the rats were allowed to acclimatize for 5 days before assignment to the experimental groups. During this period, animals were identified by tail marking towards tip of the tail, using a non-toxic marker pen. Animals were observed minimum once daily for clinical signs and twice daily for morbidity and mortality.

#### 2.4.2 Randomization of animals

A detailed clinical examination was performed before randomization, following which, 30 male and 30 female animals were randomly allocated to different groups on the basis of their body weights, as mentioned in Table 3. Each group comprised of five males and five female animals. During randomization, the animals were assigned a permanent animal number and marked towards the base of the tail using a non-toxic marker pen throughout the study period. The body weight range of the male rats at the initiation of oral administration were 212.5–250.0 g, whereas the range for female animals were 187.0–228.0 g. At the commencement of vehicle/DSV administration, the weight variation of animals did not exceed ±20% of the mean weight of each sex. The animals not selected for study following randomization were returned to the facility without any further investigations. Following randomization, ophthalmoscopic examination was performed using an ophthalmoscope (HEINE Optotechnik, Germany) by a trained veterinarian.

**TABLE 3:** Experimental design.

Group no.	Group	Treatment	Number of animals	Dose of DSV (mg/kg/day)	Concentration of formulation prepared (mg/mL)
28-day Treatment Group					
G1	Control	Vehicle	5	0	0
G2	Low dose	DSV	5	100	10
G3	Mid dose		5	300	30
G4	High dose		5	1,000	100
14-day Recovery Group					
G1R	Control	Vehicle	5	0	0
G4R	High dose	DSV	5	1,000	100

#### 2.4.3 Preparation of the formulations of DSV and their oral administration

Required quantity of DSV was weighed on an analytical balance (Contech, India) and transferred to a mortar. DSV was then triturated well with a pestle. Subsequently, a small volume of 0.5% Methyl Cellulose (Loba Chemie Private Limited, India) solution was added by using a syringe with continuous stirring. After DSV was properly wetted, remaining volume of methylcellulose was added dropwise by using a syringe, with continuous stirring to obtain a stable suspension. All formulations were prepared fresh, on each day of DSV administration. Animals from group G2 (low dose), G3 (mid dose), G4 (high dose) and G4R (high dose) received DSV at the doses of 100, 300, 1,000 and 1,000 mg/kg body weight, respectively by gavage. Control (G1) and Control Recovery (G1R) group animals were administered the vehicle alone by oral route and received the highest dose volume based on dose volume of high dose group. The dose volume administered to the animals was fixed at 10 mL/kg body weight.

#### 2.4.4 Study observations

##### 2.4.4.1 Mortality and clinical signs

All the animals were observed carefully once a day for clinical signs, and at least twice a day for morbidity and mortality, throughout the experimental duration. Following treatment cessation, observations were continued for an additional 14 days for control-recovery (G1R) and high-dose-recovery (G4R) groups.

##### 2.4.4.2 Detailed clinical observations

Animals from all the groups were observed for detailed clinical observations once in week up to study termination. The assessments included, alteration in skin, fur, eyes, and mucous membranes, incidence of secretions and excretions and fundamental observations of autonomic activity (e.g., lacrimation, piloerection, pupil size, and unusual respiratory pattern) (Organization for Economic Co-Operation and Development, 1995).

##### 2.4.4.3 Body weight recording

Body weights were recorded on Days 1, 8, 15, 22 and 28 for main group and continued on days 35 and 42 for the animals of the recovery group by employing a pan balance (Contech, India). Additionally, body weights were also recorded before randomization and on the day of necropsy.

##### 2.4.4.4 Food consumption recording

Food consumption of all the groups were recorded by using a pan balance (Contech, India) once a week till the end of the observation period. The quantity of food consumed by each animal per cage in week was calculated using the following formula:

$$Food\;consumption\;per\;week\;\left(g\right)=\frac{Food\;offered\;\left(g\right)-Food\;leftover\;\left(g\right)}{Number\;of\;animals}$$

##### 2.4.4.5 Ophthalmoscopic examination

Animals were subjected to ophthalmoscopic examination by employing an ophthalmoscope (HEINE Optotechnik, Germany) during the last week of treatment for vehicle control (G1) and high dose (G4) group animals. The results of examinations during last week did not reveal any treatment-related ocular changes, hence the examination was not extended to lower dose groups. Animals were subjected to ophthalmoscopic examination after inducing mydriasis with 1% Tropicamide (Sunways India Private Limited, India).

##### 2.4.4.6 Clinical pathology observations

After completion of the vehicle/DSV administration period on day 28, all the main group animals and after completion of recovery period at day 42, all the recovery group animals were fasted overnight. The blood samples were withdrawn from retro-orbital plexus under mild anaesthesia on day 29 from main study groups and on day 43 from recovery group animals for hematological, coagulation and clinical chemistry analysis.

###### 2.4.4.6.1 Hematology and coagulation parameters

For hematology analysis, the blood withdrawn from the animals was directly dispensed in centrifuge tubes that contained ethylenediaminetetraacetic acid dipotassium salt (EDTA 2K, HIMEDIA, India) as an anticoagulant. Thereafter, the complete blood count was enumerated by aspirating the blood in a Sysmex XP-100, a 3-part automated hematology analyzer (Sysmex Corporation, Japan). The parameters measured included, Red Blood Cell count (RBC), Hematocrit (HCT), Mean Corpuscular Volume (MCV), Hemoglobin Concentration (HGB), Mean Corpuscular Hemoglobin (MCH), Mean Corpuscular Hemoglobin Concentration (MCHC), Platelet Count (PLT), White Blood Cell count (WBC). Reticulocytes (RET) and differential leukocyte counts were enumerated manually after staining the slides with New Methylene Blue and Giemsa stains, respectively. For coagulation analysis, whole blood was collected in vials containing Sodium Citrate (Merck KGaA, Germany) and the coagulation parameters: Prothrombin Time (PT) and Activated Partial Thromboplastin Time (APTT) were measured by using Erba ECL-105 Coagulation Analyzer (Erba Mannheim, Germany).

###### 2.4.4.6.2 Clinical chemistry evaluation

Blood samples were collected into vials containing heparin (Samarth Life Sciences Private Limited, India) for plasma and without anticoagulant for serum separation, respectively. Plasma and sera were separated by centrifugation at 3,000 rpm for 10–15 min, respectively. Serum samples were used for electrolyte analysis, whereas plasma samples were processed for clinical chemistry analysis. Serum samples were processed using Sensacore Electrolyte Analyzer ST-200CL (Sensacore, India), whereas plasma samples were processed using Erba EM Destiny 180 Auto Analyzer (Erba Mannheim, Germany). The following clinical chemistry parameters were evaluated: Glutamate Oxaloacetate Transaminase (GOT or AST), Glutamate Pyruvate Transaminase (GPT or ALT), Alkaline Phosphatase (ALP), Total Bilirubin (BILT), Blood Urea Level (BUL), calculated Blood Urea Nitrogen (BUN), Creatinine (CREAT), Glucose (GLU), Total Cholesterol (CHOLE), Total Protein (PRO), Albumin (ALB), Protein: Albumin ratio (PAR), Sodium (Na^+^), Potassium (K^+^) and Chloride (Cl^−^).

###### 2.4.4.6.3 Urinalysis

During the last week of treatment and recovery periods, all the animals from each group were subjected to overnight urine collection by utilizing metabolic cages. Urine samples were analyzed for their appearance (colour and clarity). Then, bilirubin (BIL), glucose (GLU), protein (PRO), pH, and specific gravity (SG) were qualitatively estimated by using Erba LAURA SMART urine strip analyzer (Erba Mannheim, Germany).

##### 2.4.4.7 Necropsy and gross pathological observation

On day 29, all animals from main group and on day 43, all animals from recovery group were humanely sacrificed by inhalation of carbon dioxide. All the animals were subjected to detailed gross pathological examination, which encompassed a careful examination of the external surface of the body, all orifices, and the cranial, thoracic and abdominal cavities with their contents (Organization for Economic Co-Operation and Development, 1995).

##### 2.4.4.8 Harvesting of organs and organ weight recording

After gross pathology examination, liver, kidneys, adrenals, testes/ovaries, thymus, spleen, brain and heart of all animals were trimmed off to remove any adherent tissue and were weighed wet. Paired organs were weighed together on a weighing balance (Contech, India). Relative organ weights were calculated for each animal by using following formula:

$$Relative\;organ\;weight\;\left(g\right)=\frac{Organ\;weight\;\left(g\right)}{Terminal\;body\;weight\;\left(g\right)}\times 100$$

The following organs/tissues of all animals were collected at necropsy and fixed in 10% neutral buffered formalin for histopathological examination: adrenals, aorta, bone (bone marrow), cerebrum, cerebellum, midbrain, epididymis, esophagus, heart, kidneys, large intestine, liver, lungs, lymph nodes (mesenteric), ovaries, pancreas, sciatic nerve, pituitary, seminal vesicles, prostate, skeletal muscle, skin, small intestine, spleen, stomach, spinal cord, thymus, thyroid and parathyroid, trachea, urinary bladder, vagina, uterus and mammary gland. Modified Davidson’s Fixative was used for the fixation of the eyes and the testes.

##### 2.4.4.9 Histological evaluation

The samples of all the preserved organs and tissues from the animals allocated to the control (G1) and high dose group of DSV(G4) were processed routinely and embedded in paraffin by employing a Tissue Embedding Station (Especialidades Médicas MYR, S.L., Spain). The sections of 3–5 µm thickness were prepared by utilizing CUT 5062 microtome (SLEE medical GmbH, Germany), stained with hematoxylin-eosin (Merck KGaA, Germany) and examined microscopically by utilizing an LX 300 microscope (Labo America, Inc., United States).

### 2.5 Data analysis

Data was compiled from all the study groups and mean and standard deviation were computed. The body weight, feed consumption, hematology, clinical chemistry, urinalysis (for specific gravity and urinary pH) and relative organ weight data was analyzed using one-way ANOVA followed by Dunnett’s *post hoc* multiple comparison test for main groups, and Student’s-test for recovery groups using Graph Pad Prism Version 7.03 (GraphPad Inc., San Diego, CA, United States). All analysis and comparisons were evaluated at the 5% (P < 0.05) level in comparison with the respective controls.

## 3 Results

### 3.1 Mortality and clinical signs

As evidenced by the daily examinations, no mortality, morbidity, or abnormal clinical signs, attributable to DSV manifested in any of the rats from both the main and recovery groups, throughout the study duration (Supplementary Table S1). Additionally, weekly detailed clinical examinations did not reveal any clinical abnormalities related to DSV, in any animal during the entire course of the study (Supplementary Table S2).

### 3.2 Effects of DSV on body weights and food intake

**FIGURE 1:**
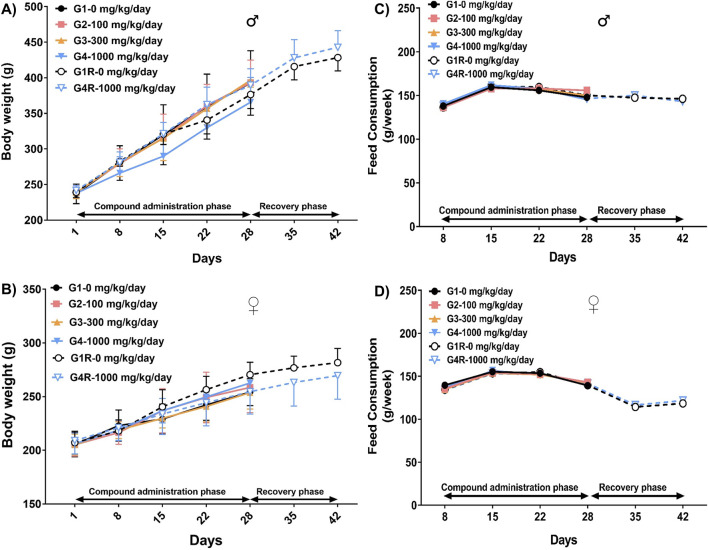
Body weight (Mean ± Standard Deviation) and food consumption (Mean) of male rats [**(A,C)**, respectively] and female rats [**(B,D)**, respectively], that orally received DSV at the doses of 0, 100, 300 and 1,000 mg/kg/day, for 28 consecutive days followed by a 14-day treatment-free recovery period as described in the methods section.

When compared to their respective controls, no statistically significant changes were noted in the body weights of the animals administered with DSV in both the main and the recovery groups, throughout the experimental period (Figures 1A,C). Likewise, the weekly food intake in the animals from the DSV-administered groups was comparable to that of their respective control groups (Figures 1B,D).

### 3.3 Ocular effects of DSV

The animals assigned to the control and high dose of DSV (1,000 mg/kg/day) groups in the main study were examined for abnormal ophthalmic signs on day 28. No DSV-related ophthalmic anomaly was detected in any animal on the scheduled assessment day (Supplementary Table S3). Since no aberration was observed in any animal administered with the high dose of DSV, the rats which received the low and mid-dose of the calcio-herbal medicine, were not subjected to ophthalmoscopic examination.

### 3.4 Effect of DSV on hematological parameters

The hematological parameters in the rats administered with DSV, from both the main as well as the recovery groups, were largely comparable to their respective controls (Tables 4, 5). However, amongst the animals allocated to the main study groups, a statistically significant increase in total erythrocyte count, hemoglobin concentration and hematocrit (p < 0.05; Table 5) was noted in female rats, which were administered the mid-dose of DSV (300 mg/kg/day), when compared to the control group. This effect, however, cannot be directly attributed to DSV, due to an absence of an explicit relation to the doses administered. Accordingly, it could be categorized as an incidental finding. Moreover, the observed values were within the historical ranges for SD Rats at the site of the study.

**TABLE 4:** Hematological parameters and coagulation analysis in male rats.

Parameter	28-day treatment of DSV (mg/kg/day)				14-day recovery (mg/kg/day)	
	G1 (0)	G2 (100)	G3 (300)	G4 (1,000)	G1R (0)	G4R (1,000)
RBC (10^6^/µL)	7.45 ± 0.19	7.22 ± 0.30	7.35 ± 0.34	7.49 ± 0.21	7.27 ± 0.42	7.55 ± 0.28
HGB (g/dL)	14.58 ± 0.33	14.28 ± 0.78	14.86 ± 0.40	14.78 ± 0.45	13.90 ± 0.56	14.30 ± 0.31
HCT (%)	44.48 ± 1.35	43.30 ± 2.57	44.76 ± 2.48	44.52 ± 1.24	42.56 ± 2.28	44.40 ± 1.45
MCV (fL)	59.76 ± 1.47	59.96 ± 1.40	60.88 ± 1.35	59.48 ± 0.83	58.56 ± 1.06	58.82 ± 1.22
MCH (pg)	19.58 ± 0.40	19.80 ± 0.53	20.22 ± 0.68	19.76 ± 0.44	19.14 ± 0.53	17.76 ± 2.52
MCHC (g/dL)	32.82 ± 0.74	33.00 ± 0.41	33.22 ± 1.07	33.22 ± 0.87	32.70 ± 0.63	32.18 ± 0.47
PLT (10^3^/µL)	990.40 ± 90.95	1,028.00 ± 130.39	911.40 ± 90.78	917.40 ± 224.72	1,023.20 ± 147.64	1,030.20 ± 176.73
RET (%)	1.36 ± 0.17	1.44 ± 0.17	1.36 ± 0.17	1.40 ± 0.20	1.48 ± 0.11	1.44 ± 0.17
WBC (10^3^/µL)	10.08 ± 1.96	12.68 ± 3.75	7.26 ± 2.25	9.84 ± 4.44	12.14 ± 3.85	12.56 ± 0.74
NEU (%)	26.20 ± 2.39	25.40 ± 1.95	25.60 ± 2.41	26.80 ± 2.28	26.60 ± 1.14	25.40 ± 3.65
LYM (%)	70.80 ± 2.59	72.40 ± 1.14	72.20 ± 2.17	71.60 ± 2.41	71.00 ± 1.58	72.80 ± 3.42
MONO (%)	1.00 ± 0.00	0.60 ± 0.55	0.80 ± 0.45	0.60 ± 0.55	0.80 ± 0.45	0.40 ± 0.55
EOS (%)	1.80 ± 0.84	1.40 ± 1.14	1.20 ± 1.10	0.80 ± 0.84	1.40 ± 0.55	1.20 ± 1.10
BASO (%)	0.20 ± 0.45	0.20 ± 0.45	0.20 ± 0.45	0.20 ± 0.45	0.20 ± 0.45	0.20 ± 0.45
PT (sec)	17.16 ± 1.03	17.13 ± 3.03	15.94 ± 2.42	15.72 ± 2.02	16.62 ± 1.27	16.17 ± 0.67
APTT (sec)	18.36 ± 1.42	19.06 ± 0.93	19.82 ± 0.65	20.26 ± 0.54^*^	18.78 ± 1.55	19.64 ± 3.17

Data is presented as Mean ± Standard Deviation (n = 5 animals per group). *p < 0.05 vs. G1 (One-Way ANOVA, followed by Dunnett’s *post hoc* multiple comparison test). RBC, red blood cell; HGB, hemoglobin; HCT, hematocrit; MCV, mean corpuscular volume; MCH, mean corpuscular hemoglobin; MCHC, mean corpuscular hemoglobin concentration; PLT, platelet; RET, reticulocyte; WBC, white blood cell; NEU, neutrophils; LYM, lymphocytes; MONO, monocytes; EOS, eosinophils; BASO, basophils; PT, prothrombin time; APTT, activated partial thromboplastin time.

**TABLE 5:** Hematological parameters and coagulation analysis in female rats.

Parameter	28-day treatment of DSV (mg/kg/day)				14-day recovery (mg/kg/day)	
	G1 (0)	G2 (100)	G3 (300)	G4 (1,000)	G1R (0)	G4R (1,000)
RBC (10^6^/µL)	6.61 ± 0.29	6.90 ± 0.12	7.09 ± 0.08^*^	6.87 ± 0.28	6.78 ± 0.32	6.84 ± 0.39
HGB (g/dL)	12.98 ± 0.70	13.52 ± 0.28	14.44 ± 0.65^*^	13.86 ± 0.43	13.02 ± 0.55	13.20 ± 0.52
HCT (%)	37.82 ± 2.37	39.40 ± 0.83	42.12 ± 2.40^*^	39.88 ± 1.06	38.88 ± 1.51	39.74 ± 2.24
MCV (fL)	57.20 ± 2.10	57.10 ± 0.44	59.38 ± 2.78	58.06 ± 1.13	57.40 ± 0.87	58.18 ± 1.71
MCH (pg)	19.62 ± 0.71	19.60 ± 0.35	20.34 ± 0.72	20.18 ± 0.53	19.22 ± 0.38	19.34 ± 0.56
MCHC (g/dL)	34.34 ± 0.52	34.32 ± 0.41	34.30 ± 0.45	34.74 ± 0.55	33.48 ± 0.65	33.48 ± 0.46
PLT (10^3^/µL)	1,094.00 ± 134.73	1,063.00 ± 106.00	1,087.40 ± 105.45	1,074.80 ± 109.90	1,017.80 ± 302.98	1,076.20 ± 145.35
RET (%)	1.44 ± 0.17	1.48 ± 0.11	1.40 ± 0.20	1.36 ± 0.17	1.40 ± 0.14	1.48 ± 0.11
WBC (10^3^/µL)	6.62 ± 2.64	8.54 ± 3.94	6.88 ± 2.07	8.04 ± 2.65	6.70 ± 1.54	7.48 ± 4.84
NEU (%)	27.20 ± 1.92	26.20 ± 1.92	27.00 ± 1.87	26.40 ± 1.67	27.60 ± 1.14	27.00 ± 1.58
LYM (%)	70.60 ± 2.30	71.00 ± 2.00	70.40 ± 1.82	71.00 ± 1.58	69.40 ± 1.82	70.20 ± 1.48
MONO (%)	0.80 ± 0.45	0.80 ± 0.45	0.80 ± 0.45	0.80 ± 0.45	0.80 ± 0.45	0.60 ± 0.55
EOS (%)	1.40 ± 0.89	1.80 ± 0.84	1.80 ± 1.10	1.60 ± 1.14	2.00 ± 0.71	2.00 ± 0.71
BASO (%)	0.00 ± 0.00	0.20 ± 0.45	0.00 ± 0.00	0.20 ± 0.45	0.20 ± 0.45	0.20 ± 0.45
PT (sec)	17.36 ± 1.06	16.00 ± 1.99	15.12 ± 1.31	17.48 ± 2.10	16.46 ± 1.76	16.19 ± 1.40
APTT (sec)	19.76 ± 1.24	19.30 ± 0.93	20.72 ± 0.69	20.86 ± 0.71	17.18 ± 3.39	21.04 ± 1.42^#^

Data is presented as Mean ± Standard Deviation (n = 5 animals per group). *, p < 0.05 vs. G1 (One-Way ANOVA, followed by Dunnett’s *post hoc* multiple comparison test), #, p < 0.05 vs. G1R (Student’s t-test). RBC, red blood cell; HGB, hemoglobin; HCT, hematocrit; MCV, mean corpuscular volume; MCH, mean corpuscular hemoglobin; MCHC, mean corpuscular hemoglobin concentration; PLT, platelet; RET, reticulocyte; WBC, white blood cell; NEU, neutrophils; LYM, lymphocytes; MONO, monocytes; EOS, eosinophils; BASO, basophils; PT, prothrombin time; APTT, active partial thromboplastin time.

### 3.5 Effects of DSV on coagulation parameters

The effect of DSV on prothrombin time and activated partial thromboplastin time was evaluated in this study, wherein, both the parameters were, for the most part, not affected by DSV administration in animals from both the main and recovery groups (Tables 4, 5). Nevertheless, as compared to the control group, a statistically significant increase in activated partial thromboplastin time (p < 0.05; Table 4) was observed in male rats from the main study group, which were administered the high dose of DSV (1,000 mg/kg/day). A similar outcome was additionally observed in the female rats (p < 0.05; Table 5) in the recovery group, that were administered the high dose of DSV (1,000 mg/kg/day). This increase in the measure of the intrinsic pathway of the hemostatic mechanism was rather minor and was well within the normal range defined for SD rats at the site of the study. Consequently, this observation is unlikely to be of any clinical significance.

### 3.6 Effect of DSV on clinical chemistry parameters

Administration of DSV did not alter the clinical chemistry parameters of the rats of either sex assigned to the main and the recovery groups (Tables 6, 7).

**TABLE 6:** Clinical chemistry values in male rats.

Parameter	28-day treatment of DSV (mg/kg/day)				14-day recovery (mg/kg/day)	
	G1 (0)	G2 (100)	G3 (300)	G4 (1,000)	G1R (0)	G4R (1,000)
GPT (U/L)	151.98 ± 14.04	141.72 ± 24.13	126.80 ± 30.55	127.26 ± 20.45	102.90 ± 17.45	107.10 ± 13.74
GOT (U/L)	134.04 ± 67.43	121.94 ± 32.01	127.42 ± 34.75	117.66 ± 34.30	121.36 ± 22.94	99.98 ± 31.09
BUL (mg/dL)	15.32 ± 13.39	17.10 ± 6.67	14.94 ± 6.61	13.90 ± 6.86	16.50 ± 6.26	16.42 ± 4.73
BUN (mg/dL)	7.20 ± 6.14	8.00 ± 3.32	7.00 ± 3.32	6.60 ± 3.13	7.60 ± 2.88	7.80 ± 2.17
CREAT (mg/dL)	0.69 ± 0.09	0.70 ± 0.06	0.70 ± 0.09	0.69 ± 0.02	0.70 ± 0.15	0.71 ± 0.08
GLU (mg/dL)	105.30 ± 6.24	105.62 ± 11.14	108.64 ± 17.46	104.38 ± 6.05	107.54 ± 15.76	102.12 ± 13.48
CHOLE (mg/dL)	76.40 ± 8.62	73.80 ± 12.21	89.20 ± 14.13	67.40 ± 19.42	84.20 ± 13.68	76.20 ± 20.45
ALB (g/dL)	3.47 ± 0.57	3.54 ± 0.47	3.49 ± 0.56	3.62 ± 0.47	3.34 ± 0.69	3.35 ± 1.41
PRO (g/dL)	6.03 ± 0.22	6.05 ± 0.09	6.03 ± 0.16	6.06 ± 0.17	6.10 ± 0.25	6.04 ± 0.17
PAR	1.78 ± 0.31	1.74 ± 0.25	1.76 ± 0.27	1.70 ± 0.27	1.90 ± 0.43	2.03 ± 0.69
ALP (U/L)	176.00 ± 49.87	161.60 ± 116.04	139.80 ± 24.99	107.80 ± 48.31	124.80 ± 42.61	125.60 ± 67.14
BILT (mg/dL)	0.38 ± 0.05	0.40 ± 0.05	0.36 ± 0.09	0.37 ± 0.05	0.33 ± 0.05	0.39 ± 0.07
Na^+^ (mmol/L)	146.36 ± 1.68	144.54 ± 0.48	145.74 ± 0.71	149.46 ± 7.81	143.94 ± 1.08	143.40 ± 1.13
K^+^ (mmol/L)	6.03 ± 0.47	6.22 ± 0.29	5.46 ± 0.63	5.21 ± 1.02	6.63 ± 0.47	6.31 ± 0.30
Cl^−^ (mmol/L)	83.88 ± 40.19	101.44 ± 0.92	101.58 ± 1.16	105.86 ± 2.34	102.76 ± 1.70	102.48 ± 1.75

Data is presented as Mean ± Standard Deviation (n = 5 animals per group). GPT, glutamate pyruvate transaminase; GOT, glutamate oxaloacetate transaminase; BUL, blood urea level; BUN, blood urea nitrogen; CREAT, creatinine; GLU, glucose; CHOLE, total cholesterol; ALB, albumin; PRO, total protein; PAR, protein: albumin Ratio; BILT, total bilirubin.

**TABLE 7:** Clinical chemistry values in female rats.

Parameter	28-day treatment of DSV (mg/kg/day)				14-day recovery (mg/kg/day)	
	G1 (0)	G2 (100)	G3 (300)	G4 (1,000)	G1R (0)	G4R (1,000)
GPT (U/L)	93.94 ± 27.35	95.00 ± 31.96	88.04 ± 17.20	84.32 ± 20.40	79.52 ± 12.86	95.48 ± 13.07
GOT (U/L)	103.74 ± 21.65	94.26 ± 27.35	82.90 ± 10.16	86.30 ± 18.91	105.68 ± 13.79	107.16 ± 20.30
BUL (mg/dL)	10.80 ± 3.35	14.18 ± 7.41	12.78 ± 5.02	10.04 ± 3.16	12.50 ± 1.58	13.32 ± 1.52
BUN (mg/dL)	5.00 ± 1.41	6.60 ± 3.44	5.80 ± 2.17	4.40 ± 1.52	5.80 ± 0.84	6.00 ± 0.71
CREAT (mg/dL)	0.58 ± 0.06	0.57 ± 0.22	0.58 ± 0.06	0.59 ± 0.06	0.59 ± 0.09	0.58 ± 0.07
GLU (mg/dL)	104.06 ± 10.24	104.62 ± 13.60	105.90 ± 11.36	100.60 ± 10.32	104.90 ± 8.91	112.60 ± 11.71
CHOLE (mg/dL)	89.40 ± 18.56	74.40 ± 21.87	92.20 ± 13.59	80.40 ± 22.69	93.20 ± 20.73	94.40 ± 10.45
ALB (g/dL)	3.35 ± 0.47	3.37 ± 0.42	3.44 ± 0.37	3.39 ± 0.32	3.36 ± 0.24	3.31 ± 0.11
PRO (g/dL)	6.41 ± 0.52	6.51 ± 0.52	6.50 ± 0.93	6.87 ± 0.16	6.14 ± 0.42	6.52 ± 0.25
PAR	1.94 ± 0.30	1.94 ± 0.16	1.89 ± 0.26	2.04 ± 0.19	1.83 ± 0.16	1.97 ± 0.06
ALP (U/L)	98.40 ± 48.99	74.40 ± 11.72	76.40 ± 25.56	70.60 ± 49.16	147.40 ± 71.52	136.20 ± 29.01
BILT (mg/dL)	0.44 ± 0.11	0.44 ± 0.14	0.35 ± 0.04	0.42 ± 0.08	0.39 ± 0.23	0.34 ± 0.11
Na^+^ (mmol/L)	145.50 ± 1.15	145.22 ± 0.75	145.36 ± 2.32	144.56 ± 1.52	143.32 ± 2.17	144.04 ± 0.72
K^+^ (mmol/L)	5.15 ± 0.92	5.01 ± 0.22	5.26 ± 1.30	5.85 ± 1.23	5.86 ± 0.57	5.68 ± 0.40
Cl^−^ (mmol/L)	103.52 ± 1.29	103.22 ± 0.82	103.26 ± 1.46	103.26 ± 0.92	105.50 ± 2.19	103.46 ± 1.56

Data is presented as Mean ± Standard Deviation (n = 5 animals per group). GPT, glutamate pyruvate transaminase; GOT, glutamate oxaloacetate transaminase; BUL, blood urea level; BUN, calculated blood urea nitrogen; CREAT, creatinine; GLU, glucose; CHOLE, total cholesterol; ALB, albumin; PRO, total protein; PAR; protein: albumin Ratio; BILT, total bilirubin.

### 3.7 Effect of DSV on qualitative urinalysis parameters

DSV administration in rats from both the main and recovery groups, did not have a significant effect on the urinalysis parameters when compared to their respective control groups (Table 8).

**TABLE 8:** Qualitative urinalysis in rats.

Parameter	28-day treatment of DSV (mg/kg/day)				14-day recovery (mg/kg/day)	
	G1 (0)	G2 (100)	G3 (300)	G4 (1,000)	G1R (0)	G4R (1,000)
Males						
Specific Gravity	1.000 ± 0.000	1.004 ± 0.004	1.014 ± 0.004	1.016 ± 0.004	1.030 ± 0.000	1.030 ± 0.000
pH	5.90 ± 1.34	6.50 ± 0.00	6.30 ± 0.27	6.10 ± 0.22	6.50 ± 0.00	6.50 ± 0.00
Colour: Yellow	5/5	5/5	5/5	5/5	5/5	5/5
Turbidity: Clear	5/5	5/5	5/5	5/5	5/5	5/5
Bilirubin						
a) Negative	5/5	5/5	5/5	5/5	5/5	5/5
b) Positive	0/5	0/5	0/5	0/5	0/5	0/5
Protein						
a) Negative	5/5	5/5	5/5	5/5	5/5	5/5
b) Positive	0/5	0/5	0/5	0/5	0/5	0/5
Glucose						
a) Negative	5/5	5/5	5/5	5/5	5/5	5/5
b) Positive	0/5	0/5	0/5	0/5	0/5	0/5
Females						
Specific Gravity	1.009 ± 0.002	1.013 ± 0.003	1.018 ± 0.004	1.013 ± 0.007	1.011 ± 0.004	1.013 ± 0.016
pH	6.50 ± 0.00	6.20 ± 0.27	6.10 ± 0.22	7.20 ± 0.76	7.10 ± 0.55	7.20 ± 0.76
Colour: Yellow	5/5	5/5	5/5	5/5	5/5	5/5
Turbidity: Clear	5/5	5/5	5/5	5/5	5/5	5/5
Bilirubin						
a) Negative	5/5	5/5	5/5	5/5	5/5	5/5
b) Positive	0/5	0/5	0/5	0/5	0/5	0/5
Protein						
a) Negative	5/5	5/5	5/5	5/5	5/5	5/5
b) Positive	0/5	0/5	0/5	0/5	0/5	0/5
Glucose						
a) Negative	5/5	5/5	5/5	5/5	5/5	5/5
b) Positive	0/5	0/5	0/5	0/5	0/5	0/5

Data is presented as Mean ± Standard Deviation (n = 5 animals per group) for specific gravity and pH and number of animals for yellow colour, no turbidity and for the presence or absence of bilirubin, protein and glucose in the urine.

### 3.8 Relative organ weights in DSV-administered rats

The relative organ weights in rats, from the main and recovery groups, that received DSV were similar to those of their corresponding controls (Tables 9, 10).

**TABLE 9:** Relative organ weights (%) in male rats with respect to the terminal body weight.

Parameter	28-day treatment of DSV (mg/kg/day)				14-day recovery (mg/kg/day)	
	G1 (0)	G2 (100)	G3 (300)	G4 (1,000)	G1R (0)	G4R (1,000)
Body weight (g)^a^	385.10 ± 45.32	386.30 ± 31.30	389.50 ± 9.17	359.50 ± 34.35	421.40 ± 18.73	435.90 ± 23.45
Liver	3.283 ± 0.341	3.463 ± 0.125	3.082 ± 0.483	3.400 ± 0.202	3.156 ± 0.299	3.127 ± 0.498
Spleen	0.168 ± 0.012	0.180 ± 0.013	0.167 ± 0.032	0.170 ± 0.019	0.159 ± 0.020	0.167 ± 0.032
Heart	0.350 ± 0.042	0.346 ± 0.009	0.334 ± 0.012	0.354 ± 0.024	0.324 ± 0.022	0.313 ± 0.042
Thymus	0.134 ± 0.033	0.101 ± 0.033	0.089 ± 0.038	0.104 ± 0.030	0.101 ± 0.016	0.092 ± 0.013
Kidneys	0.703 ± 0.057	0.691 ± 0.035	0.644 ± 0.056	0.694 ± 0.017	0.652 ± 0.053	0.624 ± 0.071
Adrenals	0.013 ± 0.002	0.012 ± 0.002	0.013 ± 0.002	0.011 ± 0.002	0.013 ± 0.001	0.012 ± 0.002
Testes	0.646 ± 0.081	0.759 ± 0.081	0.739 ± 0.083	0.727 ± 0.138	0.652 ± 0.062	0.550 ± 0.093
Brain	0.527 ± 0.070	0.516 ± 0.058	0.506 ± 0.024	0.544 ± 0.060	0.458 ± 0.027	0.439 ± 0.045

^a^ Represents the body weight on the day of necropsy. Data is presented as Mean ± Standard Deviation (n = 5 animals per group).

**TABLE 10:** Relative organ weights (%) in female rats with respect to the terminal body weight.

Parameter	28-day treatment of DSV (mg/kg/day)				14-day recovery (mg/kg/day)	
	G1 (0)	G2 (100)	G3 (300)	G4 (1,000)	G1R (0)	G4R (1,000)
Body weight (g)^a^	247.20 ± 12.86	251.40 ± 24.24	248.20 ± 14.33	257.00 ± 5.79	272.30 ± 9.59	262.90 ± 21.11
Liver	3.112 ± 0.263	3.252 ± 0.278	3.247 ± 0.222	3.193 ± 0.423	3.499 ± 0.344	3.354 ± 0.463
Spleen	0.213 ± 0.024	0.239 ± 0.071	0.206 ± 0.027	0.206 ± 0.008	0.202 ± 0.027	0.192 ± 0.036
Heart	0.350 ± 0.026	0.353 ± 0.044	0.362 ± 0.020	0.438 ± 0.159	0.335 ± 0.022	0.352 ± 0.036
Thymus	0.113 ± 0.039	0.111 ± 0.016	0.124 ± 0.040	0.116 ± 0.043	0.133 ± 0.032	0.143 ± 0.016
Kidneys	0.699 ± 0.038	0.700 ± 0.037	0.668 ± 0.071	0.672 ± 0.077	0.647 ± 0.048	0.710 ± 0.061
Adrenals	0.023 ± 0.003	0.024 ± 0.005	0.022 ± 0.005	0.021 ± 0.004	0.022 ± 0.002	0.021 ± 0.002
Ovaries	0.048 ± 0.010	0.054 ± 0.009	0.042 ± 0.012	0.051 ± 0.005	0.048 ± 0.005	0.043 ± 0.003
Brain	0.791 ± 0.053	0.755 ± 0.087	0.750 ± 0.049	0.716 ± 0.009	0.683 ± 0.043	0.729 ± 0.058

^a^ Represents the body weight on the day of necropsy. Data is presented as Mean ± Standard Deviation (n = 5 animals per group).

### 3.9 Gross pathological findings

Gross pathological examination of the DSV-administered animals, in both the main as well as the recovery groups, did not demonstrate any lesions of pathological significance, by and large, when compared to their respective control groups (Table 11). Nevertheless, a minimum reduction in the size of the spleen was noticed in one female rat from the main study group, which was administered with the mid-dose of DSV (300 mg/kg/day). Based on the gross pathological observation, the spleen from the animal in question was subjected to histopathological analysis, wherein, no aberrant changes were detected. Accordingly, this observation could be regarded to be an incidental one.

**TABLE 11:** Gross pathology observations at necropsy.

Sex/Organ/Finding	28-day treatment of DSV (mg/kg/day)				14-day recovery (mg/kg/day)	
	G1 (0)	G2 (100)	G3 (300)	G4 (1,000)	G1R (0)	G4R (1,000)
Males						
NAD	5/5	5/5	5/5	5/5	5/5	5/5
Females						
Spleen						
Minimal reduction in size	0/5	0/5	1/5	0/5	0/5	0/5

### 3.10 Histopathological analysis

Histopathological examinations were conducted in animals of the main study group which received the vehicle and the high dose of DSV (1,000 mg/kg/day). When compared to the control group, the animals which received the high dose of DSV, did not reveal any abnormal histopathological alterations that could be directly attributed to the calcio-herbal medicine, DSV (Table 12).

**TABLE 12:** Histopathological observations.

Organ/Finding/Severity	Males		Females	
	28-day treatment of DSV (mg/kg/day)		28-day treatment of DSV (mg/kg/day)	
	G1 (0)	G4 (1,000)	G1 (0)	G4 (1,000)
Lungs				
*Perivascular edema and MNC infiltration*				
Minimal focal	2/5	1/5	2/5	3/5
Liver				
*Biliary hyperplasia*				
Minimal focal	1/5	2/5	2/5	1/5
*Necrotic foci*				
Minimal Focal	1/5	2/5	1/5	0/5
Kidney				
*Tubular degeneration*				
Minimal focal	1/5	0/5	1/5	1/5
Minimal multifocal	2/5	2/5	1/5	1/5
*Cyst, MNC infiltration*				
Minimal focal	1/5	1/5	1/5	1/5
Heart				
*Congestion*				
Minimal focal	1/5	1/5	1/5	1/5
Spleen				
*Congestion*				
Minimal focal	1/5	0/5	1/5	1/5

## 4 Discussion

The botanical drugs and mineral ashes present in DSV have been traditionally used in Ayurveda for the treatment of the symptoms associated with allergic as well as obstructive airway disorders, due to their anti-inflammatory, anti-oxidant and bronchospasmolytic activities. More recently DSV, has been reported to mitigate the inflammatory damage triggered in zebrafish by the injection of the recombinant spike protein of SARS-CoV2 virus (Balkrishna et al., 2020). Owing to its established *in-vivo* anti-inflammatory effects, DSV has the potential to treat the underlying airway inflammation and the ensuing airway obstruction, which is the hallmark symptom of patients afflicted with COVID-19 and consequently, DSV is a candidate for a comprehensive non-clinical and clinical investigation. Accordingly, with an aim to support the future clinical studies, we assessed the non-clinical safety of DSV, subsequent to the its repeated oral administration in male and female Sprague Dawley rats, for a period 28-consecutive days, along with a 14-day treatment-free recovery period.

While the subacute toxicity evaluation of DSV in the present study did reveal statistically significant alterations in certain parameters, however, those were not considered to be adverse and moreover not directly ascribed to DSV administration, owing to either an absence of an obvious dose-relationship or due to the lack of corresponding changes in the gross organ pathology or histopathological parameters. Moreover, the investigated parameters in which the significant changes were demonstrated were in the normal reference ranges described for Sprague Dawley rats at the site of the study. Therefore, as deduced from the study, the NOAEL for DSV is 1,000 mg/kg/day. The previously ascertained preclinical *in-vivo* pharmacological effects of DSV (Balkrishna et al., 2020), and an acceptable safety demonstrated in the present study support the further non-clinical and clinical evaluation of DSV.

In the public domain, there is a paucity of the toxicity evaluation studies of the individual botanical drugs and mineral ashes present in DSV and wherever available, only the aqueous and/or alcoholic extracts have been assessed. As a corollary, the extracts are certain to have concentrated metabolites as opposed to the powdered botanical drugs. Subsequently, the results of the toxicity studies employing the extracts cannot be extrapolated to those utilizing the raw material as such*.* Nevertheless, it is still imperative to briefly outline the available toxicological profile of the individual botanical drugs and mineral ashes present in DSV.

In DSV, the botanical drugs present in the highest proportions are *G. glabra* L., *P. chinensis* subsp. *Integerrima* (J. L. Stewart) Rech. f., and *C. cretica* L. Based on their proportion in DSV, the daily doses of these botanical drugs, which the animals would have received in our study could be calculated to 13, 39 and 130 mg/kg/day, respectively. Of these botanical drugs, the subacute toxicity of the aqueous extract of *G. glabra* L. root was evaluated in rats, wherein the animals were administered the extract by oral route at the dose levels of 100, 250 and 500 mg/kg/day for a period of 15-consecutive days (Al-Qarawi et al., 2002). A significant finding from this study was a suppressive effect of the extract on the adrenal-kidney-pituitary axis, reflected by a dose-related decrease in the plasma concentrations of cortisol, adrenocorticotrophic hormone, aldosterone and potassium, and a dose-related increase in the levels of renin and sodium. These effects were significant even at the lowest tested dose, i.e. 100 mg/kg. Nevertheless, in our study, DSV administration had no effect on the serum levels of sodium and potassium, reinforcing our finding for a lack of correlation between the toxicological effects of the extracts *vis à vis* the dried whole botanical drugs. The sub-acute toxicity of *P. chinensis* subsp. *Integerrima* (J. L. Stewart) Rech. f., has also been assessed in rats subsequent to the oral administration of a methanolic extract at the dose levels of 250, 500 and 1,000 mg/kg/day, for 28 days (Sharwan et al., 2016). In this study, no clinically relevant toxicological effects attributable to the extract were observed up to the dose level of 1,000 mg/kg, ascertaining its safety upon subacute administration. With regards to *C. cretica* L., in an *in-vivo* pharmacology study, the methanolic extract of the whole plant was administered orally for 60 days in male rats at the dose of 100 mg/kg/day, wherein it demonstrated anti-fertility activity by significantly lowering testosterone production and altering spermatogenic activity (Gupta et al., 2006). The extract of *C. cretica* L., significantly decreased the weights of the testes, epididymis, seminal vesicle and the ventral prostate. Contrastingly, in our study, we did not observe any alteration in the relative organ weight of the testes in any of the evaluated doses. Moreover, gross and histological observations of the testes, prostate, epididymis and seminal vesicle did not reveal any differences between the control animals and the rats that received the high dose of DSV. In our study, we have employed only the powdered fruit of the botanical drug as opposed to the whole plant extract. Hence, in all probability, DSV is unlikely to adversely impact male fertility.

DSV additionally contains, in equal proportions, the powdered rhizome of *Z. officinale* Roscoe, and the dried fruits of *P. longum* L. and *P. nigrum* L. In our studies, the doses of the powdered botanical drugs which would have been daily administered to the animals could be computed to 8.5, 25.5 and 85 mg/kg/day. Of these botanical drugs, the sub-acute and chronic toxicity studies of a standardized ethanolic extract of *Z. officinale* Roscoe is reported in rats (Plengsuriyakarn and Na-Bangchang, 2020). In this sub-acute toxicity study, the animals were administered the extract by oral route for 28 days at the dose levels of 500, 1,000 and 2000 mg/kg/day and the NOAEL was adjudged to be 2000 mg/kg/day. Intriguingly, the extract elicited significant and seemingly beneficial effects on the hematological parameters, namely, an increase in the red blood cell count at 2000 mg/kg/day, elevation of the platelet counts at 1,000 and 2000 mg/kg/day and increase in the total leukocyte count, neutrophil count and lymphocyte count at 500, 1,000 and 2000 mg/kg/day. Additionally, a reduction of cholesterol and triglycerides was also noted. Further, in the chronic toxicity study, the extract of *Z. officinale* Roscoe was administered to rats orally at the doses of 250, 500 and 1,000 mg/kg/day for 12 months, wherein the extract was well-tolerated up to 1,000 mg/kg/day, except for the evidence of minimal histopathological changes elicited by the mid and high dose of the extract. In case of *P. longum* L., there is a lack of data in the public domain for toxicity assessment subsequent to sub-acute administration. However, its sub-chronic toxicity evaluation has been reported, wherein, Swiss albino mice of either sex received an ethanolic extract of *P. longum* L. fruits orally at a single dose of 100 mg/kg/day for a study duration of 90 days (Shah et al., 1998). In this study, which employed a total of 60 animals (30 animals/sex), two mortalities were reported in control and three deaths occurred in treatment group. Hence, as compared to the control group this finding was not toxicologically relevant. Further, the extract-administered group showed a significant increase in the weights of the lung and the spleen. An additional outcome of the study was that *P. longum* L. fruit extract induced a statistically significant increase in the weights of the testes, caudae epididymidies and the seminal vesicles and it also elevated the sperm count as well as the sperm motility. In our study, up to the high dose of 1,000 mg/kg/day, we have neither detected any increase in the relative organ weight of the testes, nor any gross or histological alteration. Although, a dose of 100 mg/kg/day administered to mice will approximately correspond to 50 mg/kg/day for rats, it is imperative to yet again note that the toxicity evaluation in mice employed the extract whereas we have conducted our studies with the fine powder of the fruits. As is the case with *P. longum*, only the subchronic toxicity of the water extract of the fruits of *P. nigrum* L. has been described (Chunlaratthanaphorn et al., 2007), where Sprague Dawley rats of either sex were administered the extract at the doses of 300, 600 and 1,200 mg/kg/day orally for 90 days. Further, the study design also incorporated a satellite group in which animals were administered the extract at the dose level of 1,200 mg/kg/day and was maintained for a treatment-free period of an additional 28 days. In this study, the maximum tolerated dose of the extract was 1,200 mg/kg/day. This study reported, significant decreases in weights of certain organs like heart, lung, liver and kidneys. However, both the macroscopic and microscopic examination of the organs could not explicate the decrease in body weights as they did not detect any alteration as compared to the vehicle-administered animals. Further, slight but significant changes were also evident in the hematological and clinical chemistry parameters. Nevertheless, these were within the normal reference ranges reported for Sprague Dawley rats. However, in the female recovery group clearly significant increases in serum glutamate oxaloacetate transaminase was identified. In our study, however, we have not observed any effect on the relative organ weights of liver, heart and kidney and we have additionally not observed any dose-related alterations in the hematological and the clinical chemistry parameters in both the main as well as the recovery groups. Of special pertinence to our study is a sub-acute toxicity evaluation of ‘Trikatu’, an Ayurvedic formulation which comprises of the dried powders of three botanical drugs, namely, *Z. officinale* Roscoe, *P. longum* L. and *P. nigrum* L. in the ratio of 1:1:1 w/w (Chanda et al., 2009). In this study, an aqueous suspension of ‘Trikatu’ was orally administered to female Charles Foster rats for 28 days at the dose levels of 5, 50 and 300 mg/kg/day. The formulation was well tolerated up to 300 mg/kg/day and no significant changes were largely observed in most parameters studied. Nonetheless, ‘Trikatu’ administration elicited a significant increase in low density lipoprotein (LDL) at 50 and 300 mg/kg/day, a significant increase in high density lipoprotein (HDL) at 300 mg/kg/day and a significant decrease in the WBC count at 300 mg/kg/day. In DSV, as stated previously, fine powders of all the three botanical drugs are present in equal proportions and accordingly the combined doses of the three botanical drugs which the animals would have received in our study could be calculated to 26, 78 and 260 mg/kg. We have not evaluated the effect of DSV on HDL or LDL, however no significant alterations were seen in our study, on total leukocyte count even at the high dose of DSV, which corresponds to 260 mg/kg/day of the combined dosage of the three botanical drugs and is approximately equivalent to the high dose employed in the toxicological evaluation of ‘Trikatu’.

Apart from the six botanical drugs covered above, DSV additionally contains in equal proportions the fine powders of the bark of *C. verum* J. Presl, the root of *A. pyrethrum* (L.) Lag. and the flower bud of *S. aromaticum* (L.) Merr. and L. M. Perry. In our study, the doses of these powdered botanical drugs, which the animals would have received could be calculated to 6.5, 19.5 and 65 mg/kg/day. In a subchronic toxicity assessment of *C. verum* J. Presl, an ethanolic extract of the bark was orally administered to Swiss albino mice of either sex at a single dose of 100 mg/kg/day for 90 days (Shah et al., 1998). In this study employing 60 animals (30/sex), deaths occurred in two animals from the control group and three animals from the extract-administered groups. Nevertheless, the mortality was not of any toxicological relevance. Further, the mean weight of liver in the group which received the extract was significantly decreased when compared to the control group. Among the hematological parameters, hemoglobin levels in animals treated with the extract demonstrated a statistically significant decrease. As with case with *P. longum* L., the extract of *C. verum* J. Presl additionally elicited a statistically significant increase in the weights of the testes, epididymidies and the seminal vesicles. Furthermore, it also augmented the sperm count as well as the sperm motility. As stated previously, we have not detected any increase in the relative organ weight of the testes in animals administered with DSV, in addition to any corresponding macro- or microscopic findings at the high dose. Though a 100 mg/kg/day dose in mice would be equivalent to 50 mg/kg/day dose in rat, it is again pertinent to mention the usage of fine powders in our study, as opposed to the utilization of extracts, which are generally 8 to 20 times concentrated. *Anacyclus pyrethrum* (L.) Lag. Has been assessed for its subchronic toxicity potential in Wistar rats of either sex, wherein an ethanolic extract of the roots of the botanical drug were administered to the animals orally at a single dose of 1,000 mg/kg/day for 90 days (Sujith et al., 2012). In this study, no significant changes were observed as compared to the control group on body weight gain, behavioral parameters, relative organ weights, gross and histopathological, hematological as well as clinical chemistry parameters. Similar outcomes have been reported from a sub-chronic toxicity evaluation study in mice where the ethanolic extract of the root of *A. pyrethrum* (L.) Lag. was administered by oral route to mice at the dose levels of 200, 400 and 800 mg/kg/day for 90 days (Bezza et al., 2020). *Anacyclus pyrethrum* L (Lag.) root extract is thus deemed to be well tolerated at the dose of 1,000 mg/kg/day in rats, and up to the dose of 800 mg/kg/day in mice. In our study, the doses of the powdered root of the botanical drug which the animals would have received, are only a fraction of the dose of the extract evaluated in the published toxicity study. The effects of a hexane extract of the flower buds of *S. aromaticum* (L.) Merr. and L. M. Perry on the testicular function has been ascertained in male mice, at the dose of 15, 30 and 45 mg/kg/day for 35 days (Mishra and Singh, 2008). In this study, no adverse effects were seen on the body weights of animals and no impairment of liver and kidney biochemical parameters was observed. Nevertheless, 30 and 45 mg/kg/day of the extract decreased testosterone levels and testicular weight, induced degenerative alteration in the seminiferous tubules, coupled with a decrease in sperm production. With respect to our study, we did not observe any effect of DSV on the testicular weight, gross morphology and the ultrastructure of the testes. In another study, a standardized polyphenol-rich extract of the clove buds was assessed for its subacute toxicity potential, wherein Wistar rats of either sex were administered the extract at the doses of 500, 1,000 and 2,500 mg/kg/day for 28 days (Issac et al., 2015). In this reported study, the extract was not found to have any toxicological effects on body weight gain, behavior, hematological and clinical chemistry parameters up to the highest dose tested. Furthermore, subsequent to necropsy no macro- or microscopic lesions were observed in any of the evaluated organs up to the dose of 2,500 mg/kg/day.

Apart from the botanical drugs, DSV contains seven ‘*Bhasmas*’ in equal proportions. In our study the doses of the said ‘*Bhasmas*’, which the animals would have received could be calculated to 2.6, 7.8 and 26 mg/kg. Of these, one study describes the toxicity potential assessment of Mukta-Shukti Bhasma, Kapardak Bhasma and Praval Pishti (Singh et al., 2010), wherein, Charles Foster rats of either sex were daily administered the aqueous suspensions of the Bhasmas for 30 days at varying doses: Mukta-Shukti Bhasma – 25 and 85 mg/kg/day; Kapardak Bhasma and Praval Pishti – 15 and 45 mg/kg/day. These doses are nearly 3 and 10 fold of the recommended doses of the respective *Bhasmas*. In the reported study, the oral administration of the studied *Bhasmas* did not elicit any observable toxicities. In our study the doses of Mukta-Shukti *Bhasma*, Kapardak *Bhasma* and Praval Pishti which the animals are only a fraction of the highest administered doses employed in the published study. The sub-acute or sub-chronic toxicity evaluations of the other four *Bhasmas* present in DSV, namely, Abhrak *Bhasma*, Godanti *Bhasma*, Sphatika *Bhasma* and Tankan *Bhasma* are not available in the public domain. In general, however, *Bhasmas* are considered to be safe as their preparation involves a thorough purification and the preparation methods utilized. In addition, the centuries old traditional use of these classical medicines without any reported adverse effects render them suitable for human consumption.

One of the identified limitation of this non-clinical safety evaluation study, is the lack of toxicokinetic information. This experimental data is crucial for the toxicological characterization of the pharmacotherapeutic small molecules. For formulations comprising of several botanical drugs, it is relatively difficult to conduct toxicokinetics. This challenge principally arises due to the presence of complex, chemically different metabolites present in such formulations, in small quantities. Furthermore, this study reports only the non-clinical safety of DSV in rats subsequent to its repeated oral administration for a period of 28-consecutive days. Future evaluations are necessary to ascertain the subchronic and chronic preclinical safety of DSV, after its oral administration to rats for a period of 90 and 180-day, respectively. Additionally, genotoxicity and reproductive toxicity liabilities of DSV, if any, also need to be determined. Likewise, future experiments should also be directed at the assessment of the non-clinical safety of DSV in larger animals. These regulatory safety assessments would finally pave the way for the comprehensive evaluation of DSV in humans, in controlled clinical trial settings.

## 5 Conclusion

The toxicological assessment of Divya-Swasari-Vati (DSV) was conducted in accordance with OECD test guideline 407 and in compliance with OECD Principles of GLP, by orally administering it to male and female Sprague Dawley rats for 28 consecutive days, at the doses of 100, 300 and 1,000 mg/kg/day. Further, our study design also incorporated satellite groups that were either administered the vehicle, or the high dose of DSV for 28 days followed by a 14-day treatment free recovery period with an objective to ascertain the reversal, persistence or delayed occurrence of adverse effects. In our study, when compared to the vehicle-administered control group, oral administration of DSV did not cause any mortality, morbidity, abnormal clinical signs and adverse ocular effects. In addition, it did not elicit any clinically relevant alterations in the evaluated parameters with respect to body weight gain, food consumption, hematology, coagulation, urinalysis and clinical chemistry. Moreover, DSV treatment did not adversely impact the organ weights and similarly it did not lead to any gross or histopathological changes in any of the organs.

In summary, the observed non-clinical safety profile of DSV seems to be well correlated with the reported safety of its constituent botanical drugs and mineral ashes. Furthermore, based on the study outcomes, the NOAEL for DSV is adjudged to be 1,000 mg/kg in both sexes and accordingly the findings support its further non-clinical safety investigations in rodents for a longer duration and in non-rodents as well. Additionally, the study outcomes also provision for its detailed clinical evaluation in human subjects.

## Data Availability

The original contributions presented in the study are included in the article/Supplementary Material, further inquiries can be directed to the corresponding author.
