# Supplementary material for: Calcio-herbal medicine Divya-Swasari-Vati demonstrates acceptable non-clinical safety profile in the repeated-dose 28-day subacute oral toxicity study in Sprague-Dawley rats, under GLP compliance
Source: Front Pharmacol. 2025 Jun 17;16:1547532. doi: 10.3389/fphar.2025.1547532 (PMC12209181; doi:10.3389/fphar.2025.1547532)
Supplement: Supplementary file 1 [file Table1.docx]

**Supplementary Table 1.** Observation for mortality in male and female rats

| **Parameter** | **Males** | | | | | | **Females** | | | | | | |
| --- | --- | --- | --- | --- | --- | --- | --- | --- | --- | --- | --- | --- | --- |
|  | **28-day treatment of DSV**  **(mg/kg/day)** | | | | **14-day recovery (mg/kg/day)** | | **28-day treatment of DSV**  **(mg/kg/day)** | | | | | **14-day recovery (mg/kg/day)** | |
|  | **G1 (0)** | **G2 (100)** | **G3 (300)** | **G4 (1000)** | **G1R (0)** | **G4R (1000)** | **G1 (0)** | **G2 (100)** | **G3 (300)** | **G4 (1000)** | **G1R (0)** | | **G4R (1000)** |
| Mortality | 0/5 | 0/5 | 0/5 | 0/5 | 0/5 | 0/5 | 0/5 | 0/5 | 0/5 | 0/5 | 0/5 | | 0/5 |
| Survival | 5/5 | 5/5 | 5/5 | 5/5 | 5/5 | 5/5 | 5/5 | 5/5 | 5/5 | 5/5 | 5/5 | | 5/5 |

**Supplementary Table 2.** Detailed clinical observations of rats

| **Administration Days** | **28-day treatment of DSV (mg/kg/day)** | | | | **14-day recovery**  **(mg/kg/day)** | |
| --- | --- | --- | --- | --- | --- | --- |
|  | **G1 (0)** | **G2 (100)** | **G3 (300)** | **G4 (1000)** | **G1R (0)** | **G4R (1000)** |
| **Males** | | | | | | |
| 1 | NAD (5/5) | NAD (5/5) | NAD (5/5) | NAD (5/5) | NAD (5/5) | NAD (5/5) |
| 8 | NAD (5/5) | NAD (5/5) | NAD (5/5) | NAD (5/5) | NAD (5/5) | NAD (5/5) |
| 15 | NAD (5/5) | NAD (5/5) | NAD (5/5) | NAD (5/5) | NAD (5/5) | NAD (5/5) |
| 22 | NAD (5/5) | NAD (5/5) | NAD (5/5) | NAD (5/5) | NAD (5/5) | NAD (5/5) |
| 28 | NAD (5/5) | NAD (5/5) | NAD (5/5) | NAD (5/5) | NAD (5/5) | NAD (5/5) |
| 35 | NA | NA | NA | NA | NAD (5/5) | NAD (5/5) |
| 42 | NA | NA | NA | NA | NAD (5/5) | NAD (5/5) |
| **Females** | | | | | | |
| 1 | NAD (5/5) | NAD (5/5) | NAD (5/5) | NAD (5/5) | NAD (5/5) | NAD (5/5) |
| 8 | NAD (5/5) | NAD (5/5) | NAD (5/5) | NAD (5/5) | NAD (5/5) | NAD (5/5) |
| 15 | NAD (5/5) | NAD (5/5) | NAD (5/5) | NAD (5/5) | NAD (5/5) | NAD (5/5) |
| 22 | NAD (5/5) | NAD (5/5) | NAD (5/5) | NAD (5/5) | NAD (5/5) | NAD (5/5) |
| 28 | NAD (5/5) | NAD (5/5) | NAD (5/5) | NAD (5/5) | NAD (5/5) | NAD (5/5) |
| 35 | NA | NA | NA | NA | NAD (5/5) | NAD (5/5) |
| 42 | NA | NA | NA | NA | NAD (5/5) | NAD (5/5) |

NA, not applicable; NAD, no abnormality detected.

**Supplementary Table 3.** Ophthalmoscopic examination of male and female rats

| **Day** | **Dose of DSV (mg/kg/day)** | | | |
| --- | --- | --- | --- | --- |
|  | **Males** | | **Females** | |
|  | **G1 (0)** | **G4 (1000)** | **G1 (0)** | **G4 (1000)** |
| 28 | NAD (5/5) | NAD (5/5) | NAD (5/5) | NAD (5/5) |

NAD, no abnormality detected.
